# Differential gene expression profile of *Shigella dysenteriae* causing bacteremia in an immunocompromised individual

**DOI:** 10.2144/fsoa-2019-0117

**Published:** 2020-01-29

**Authors:** Dhiviya Prabaa Muthuirulandi Sethuvel, Naveen Kumar Devanga Ragupathi, Marilyn M Ninan, Joy Sarojini Michael, Shalini Anandan, Balaji Veeraraghavan

**Affiliations:** 1 Department of Clinical Microbiology, Christian Medical College, Vellore 632004, India

**Keywords:** gene expression, IcsA, invasive, RNA-Seq analysis, *Shigella*

## Abstract

**Aim:**

*Shigella* species has varying levels of virulence gene expression with respect to different sites of infection. In this study, the differential gene expression of *S. dysenteriae* in response to its site of infection was analyzed by transcriptomics.

**Methods:**

This study includes four clinical *Shigella* isolates. Transcriptomics was done for the stool and blood samples of a single patient. Isolates were screened for the presence of antimicrobial resistance genes.

**Results:**

The majority of genes involved in invasion were highly expressed in the strain isolated from the primary site of infection. Additionally, antimicrobial resistance (*dhfr*1A, *sul*II, *bla*_OXA_. *bla*_CTX-M-1_ and *qnr*S) genes were identified.

**Conclusion:**

This study provides a concise view of the transcriptional expression of clinical strains and provides a basis for future functional studies on *Shigella* spp.

## Lay abstract

*Shigella* infection is restricted to the gastrointestinal tract and rarely causes fatal extra-intestinal complications like bacteremia. There are limited studies available from India on molecular characterization of *Shigella* spp. In this study, we characterized four *Shigella* isolates obtained from bloodstream infections. *Shigella* spp. isolated from the stool and blood of one representative patient was further sequenced to study the differential gene expression profile. The differential protein expression by *S. dysenteriae* observed in this study demonstrates that it has a specific response to particular intracellular environments. Further, the *in vivo* mechanism of *Shigellae* invasion are difficult to fully study until the intracellular environment is mimicked *in vitro*. To the best of our knowledge, this is the first Indian study that compared the gene expression profile of clinical *Shigella* strains.

Diarrheal disease is the second leading cause of mortality in children according to WHO [1]. *Shigella* spp. is one of the important causes of dysentery globally and causes severe and occasionally life-threatening diarrheal infection. In Asia, it is estimated that there are 125 million infections and 14,000 deaths due to shigellosis annually [2]. Clinically, the infection may lead to rare but potentially fatal extra-intestinal complications like bacteremia. Though, bacteremia due to *Shigella* spp. is rare, it is reported in 0.4–7% of the cases. Notably, young age, malnutrition and immunosuppression are known to be the risk factors for *Shigella* spp. bacteremia [3].

Bacteria have developed various mechanisms to adhere to the organ surfaces. Some bacteria can adopt an intracellular lifestyle and get internalized inside various host cell types to replicate. Finally, pathogenic bacteria can get access to deeper tissues using various mechanisms to cross mucosal barriers and access the bloodstream, which is a gateway for all host organs [4].

Pathogens showing a variable expression of virulence factors have been observed. In fact, the expression of virulence factors depends largely on the environmental conditions. This expression of virulence genes is induced under conditions similar to those found at the site of invasion. Studies have demonstrated that a temperature of 37°C is a favorable growth condition for bacteria in intestinal epithelial cells, but bacteria grown at 30°C can be phenotypically avirulent and noninvasive [5]. The bacterium can be found either in the intestinal lumen, inside epithelial cells, phagocytes or in the bloodstream. The expression level of virulence factors in these different locations varies accordingly in order to counteract different host defense mechanisms, as reported earlier by Ribet and Cossart [4].

In this study, Shigella strains causing bacteremia were characterized using RNA-Sequencing to identify genes that are differentially expressed based on the site of infection. The genes responsible for invasion, virulence, stress, antimicrobial resistance (AMR) and other genes involved in cellular metabolism are also discussed.

## Materials & methods

### Strains

This study reports four cases of *Shigella* bacteremia diagnosed between the years 2015 and 2018. The identified isolates include two isolates each of *S. flexneri* serotype 2 and *S. dysenteriae* serotype 9. The isolates were confirmed by standard biochemical tests [6]. The isolate was serotyped using commercial antisera as per the manufacturer’s instructions (Denka Seiken, Tokyo, Japan). For transcriptome analysis, stool (FC3355) and blood (BA42767) samples of the sole patient were studied further. Patient’s symptoms, clinical diagnosis and outcome were detailed in Table 1. The term invasive (sterile site) and noninvasive (nonsterile site) refer to the pathogen isolation site in this study.

**Table 1.** Case details of Shigellemia reported in this study.

ID	Year	Species	Age/sex	Unit	GI symptoms	Clinical diagnosis	Outcome
BA12827	2015	*S. flexneri* 2	68/M	Medicine	No symptoms	DM uncontrolled	Expired
^†^BA42767 FC3355	2015	*S. dysenteriae* 9	54/M	Nephrology	Fever, loose stool, vomiting	Renal transplant on immunosuppressants	Alive
BA21871	2016	*S. dysenteriae* 9	65/M	Hematology	Acute gastroenteritis	Multiple myloma, *Shigella* septicemia	Alive
BA10746	2018	*S. flexneri* 2	27/M	Medicine	No symptoms	Presented with cognitive behavior, decreased appetite	Alive

† Isolate sequenced.

BA: Blood; DM: Diabetes mellitus; FC: Feces; GI: Gastrointestinal.

### Antimicrobial susceptibility testing

Antimicrobial susceptibility testing of isolates against ampicillin (10 μg), trimethoprim/sulphamethoxazole (1.25/23.75 μg), nalidixic acid (30 μg), ciprofloxacin (5 μg), norfloxacin (10 μg), ofloxacin (5 μg), cefpodoxime (10 μg), cefepime (30 μg), cefotaxime (30 μg), cefixime (5 μg), azithromycin (15 μg), imipenem (10 μg), meropenem (10 μg), amikacin (30 μg), gentamicin (10 μg), netilmicin (30 μg) and piperacillin/tazobactam (100/10 μg) was performed using Kirby–Bauer disc diffusion method. The results were interpreted using breakpoints recommended by the Clinical and Laboratory Standards Institute Guidelines 2018 [7]. Quality control strains used were *Escherichia coli* ATCC 35218, *Escherichia coli* ATCC 25922 and *Pseudomonas aeruginosa* ATCC 27853 for the antibiotics tested.

### AMR genes PCR

Genomic DNA was extracted using the QiaSymphony DNA extraction platform (Qiagen, Hilden, Germany). The isolates were screened for the presence of AMR (*dhfr*1A, *sul*II, *bla*_OXA_. *bla*_CTX-M-1_ and *qnr*S) genes by PCR as described earlier [8,9].

### RNA isolation

Total RNA was extracted using RNeasy Mini kit (Cat#74106, Qiagen, GmBH, Germany) according to the manufacturer’s recommendations. The RNA was checked using the Qubit^®^ 3.0 Fluorometric Quantitation kit (Invitrogen, Merelbeke, Belgium).

### RNA-sequencing & analysis

The invasive traits of selected isolates were studied by comparing the differential gene expression profile of the strain isolated exclusively from stool and blood specimen concurrently by transcriptomics. RNA-sequencing procedure was performed according to the manufacturer’s instructions using Ion Torrent (PGM) sequencer with 400-bp read chemistry (Life Technologies, CA, USA) [10]. The quality and quantity of each library was determined at each step with a Qubit^®^ 3.0 Fluorometer. *De novo* assembly using AssemblerSPAdes and annotation through RNA-Seq analysis was performed in PATRIC, the bacterial bioinformatics database and analysis resource.

### Statistical analysis

In this study, greater than twofold changes in the gene expression level between two variables was considered significant. Results were analyzed for correlation and tested for significance by Student’s t-test (p < 0.05). SPSS 16.0 and Microsoft Excel 2007 (IL, USA) were used for the statistical evaluation.

## Results & discussion

*Shigella* infection is in the majority confined to the GI tract which invades the colonic mucosa but rarely penetrates further into deeper tissues [11]. This study discusses four cases of *Shigella* bacteremia. The risk factors observed in these patients were diabetes, malignancy and immunosuppressant therapy. Previous literatures on mechanisms of pathogenesis have been described for *S. flexneri*. However, the present study has shown the invasion process of *S. dysenteriae* serotype 9.

### AMR gene PCR

Among the four isolates studied, only two isolates harbored AMR genes that codes for β-lactams, trimethoprim/sulfamethoxazole, fluroquinolones and cephalosporins, whereas no AMR genes were identified in the other two isolates. The resistance genes obtained in this study were found to be a common profile seen in the genus. The results were given in Table 2. AMR was generally more common in *Shigella* than in other enteric bacteria [12].

**Table 2.** Antimicrobial resistance profile of the study isolates.

Sample ID	Species	Resistant profile	AMR genes
BA12827	*S. flexneri* 2	R– CPD, CIP; MS - GEN, AK, P/T	*bla*_OXA_. *sul*II, *dhfr*1a, *qnr*S, *bla*_CTX-M-1_
BA42767 F3355	*S. dysenteriae* 9	R – NAL, GEN, AK	–
BA21871	*S. dysenteriae* 9	R – SXT; MS - CIP, OFL, TAX, FEP, NAL	*sul*II, *dhfr*1a, *qnr*S
BA10746	*S. flexneri* 2	R – AMP	–

AMP: Ampicillin; AK: Amikacin; BA: Blood; CIP: Ciprofloxacin; CPD: Cefpodoxime; FC: Feces; FEP: Cefepime; GEN: Gentamicin; MS: Moderate susceptible; NAL: Nalidixic acid; OFL: Ofloxacin; P/T: Piperacillin/tazobactam; R: Resistant; SXT: Trimethoprim/sulfamethoxazole; TAX: Cefotaxime.

### Differential gene expression analysis

*S. dysenteriae* serotype 9 obtained from stool and blood specimen of the single patient was studied. In RNA-Seq analysis, significant fold change was observed between non-invasive Sd_FC3355 and invasive Sd_BA42767 strains for the genes involved in invasion, virulence, motility and other cellular processes. Totally 56 genes were differentially expressed between the strains. Of these, few genes were expressed only in invasive strain Sd_BA42767 like *csp*, *dcm*, *his*E and enterotoxin genes with reduced expression, this showed the significance of these genes in the invasive phenotype of the strain. The majority of the genes (44/56 genes) were highly expressed in non-invasive isolate from the gut, which is the primary site of invasion for *Shigella* infection. Genes with no expression data were excluded from the analysis. The genes analysed were given as a supplementary material.

### Motility-associated genes

*Shigella* pathogenesis involves bacterial invasion and spread through colonic mucosa [13]. *Shigella* spp. are able to move through the cytoplasm of host cells and into adjacent cells by polymerizing actin [14] which is mediated by IcsA (virG), encoded on the 220-kb virulence plasmid [15,16]. We observed that IcsA protein was expressed only in noninvasive *Shigella* isolate (Table 3). This correlates with the fact that IcsA is required for inter- and intracellular spreading of *Shigella* within the host intestinal epithelium. *Vir*K gene, which is required for post-transcriptional regulation of *ics*A expression, has also been expressed.

**Table 3.** Gene expression profile of the two selected isolates represented in fold change.

Genes	Product	Fold change	
		Sd_FC3355	Sd_BA42767
SDY_0834/ipaH_1	Invasion plasmid antigen/internalin, putative	6	61
SDY_1062/ipaH_3	Invasion plasmid antigen/internalin, putative	2	17
SDY_2001/ipaH_4	Invasion plasmid antigen/internalin, putative	3	0
SDY_2003/ipaH_5	Invasion plasmid antigen/internalin, putative	11	0
SDY_2753/ipaH_6	Invasion plasmid antigen/internalin, putative	26	0
SDY_P003/ospB	Hypothetical protein	5	0
SDY_P004/phoN2/apy	Hypothetical protein	18	0
SDY_P010/ospD2	Enterotoxin	40	45
SDY_P023/ospD1	OspD1	361	0
SDY_P025/ipgB2	Putative chaperone (IpgB2)	1672	0
SDY_P037/ipaH4.5	Invasion plasmid antigen/internalin, putative	20	0
SDY_P038/ipaH7.8	Invasion plasmid antigen/internalin, putative	23	0
SDY_P045/ipaH1.4	Invasion plasmid antigen/internalin, putative	21	0
SDY_P055/ospC1	Hypothetical protein	15	0
SDY_P056/ospD3	Enterotoxin	16	10
SDY_P070/ospC2	Hypothetical protein	1111	0
SDY_P099/ipaH9.8	Invasion plasmid antigen/internalin, putative	17	0
SDY_P109/virK	Virulence factor VirK	365	236
SDY_P140/ipaH	Invasion plasmid antigen/internalin, putative	98	0
SDY_P151/ospC3	Hypothetical protein	224	0
SDY_P160/ipaJ	UDP-sugar hydrolase (EC 3.6.1.45); 5′-nucleotidase (EC 3.1.3.5)	1170	0
SDY_P161/virB	Chromosome (plasmid) partitioning protein ParB	76	0
SDY_P163/ipaA	Hypothetical protein	54	0
SDY_P164/ipaD	Type III secretion host injection protein (YopB)	179	0
SDY_P165/ipaC	Hypothetical protein	1244	0
SDY_P166/ipaB	Cell invasion protein SipB	1582	0
SDY_P167/ipgC	Type III secretion chaperone protein for YopD (SycD)	937	0
SDY_P169/ipgA	Chaperone ipgA	613	0
SDY_P170/icsB	Hypothetical protein	495	0
SDY_P171/ipgD	Inositol phosphate phosphatase ipgD (EC 3.1.3)	3399	0
SDY_P173/ipgF	Invasion protein IagB precursor	3235	0
SDY_P174/mxiG	Hypothetical protein	1611	0
SDY_P175/mxiH	MxiH protein	1450	0
SDY_P177/mxiJ	Type III secretion bridge between inner and outermembrane lipoprotein (YscJ, HrcJ, EscJ, PscJ)	1457	0
SDY_P179/mxiN	MxiN	1476	0
SDY_P183/mxiD	Type III secretion outermembrane pore-forming protein (YscC, MxiD, HrcC, InvG)	334	0
SDY_P184/mxiC	Type III secretion outermembrane contact-sensing protein (yopN, Yop4b, LcrE)	355	0
SDY_P185/mxiA	Type III secretion inner membrane channel protein (LcrD, HrcV, EscV, SsaV)	160	0
SDY_P186/spa15	Spa15	185	0
SDY_P187/spa47	Type III secretion cytoplasmic ATP synthase (EC 3.6.3.14, YscN, SpaL, MxiB, HrcN, EscN)	483	0
SDY_P189/spa32	Hypothetical protein	308	0
SDY_P190/spa33	Type III secretion innermembrane protein (YscQ, homologous to flagellar export components)	233	0
SDY_P191/spaP	Type III secretion innermembrane protein (YscR, SpaR, HrcR, EscR, homologous to flagellar export components); surface presentation of antigens protein SpaP	116	0
SDY_P191a/spa9	Surface presentation of antigens protein SpaQ	71	0
SDY_P192/spa29	Type III secretion innermembrane protein (YscT, HrcT, SpaR, EscT, EpaR1, homologous to flagellar export components)	28	0
SDY_P193/spa40	Type III secretion innermembrane protein (YscU, SpaS, EscU, HrcU, SsaU, homologous to flagellar export components)	33	0
SDY_P211/virA	Hypothetical protein	43	0
SDY_P214/icsA	Hypothetical protein	469	0
SDY_P224/icsP	Protease VII (Omptin) precursor (EC 3.4.23.49)	461	0
SD1617_4624/	Virulence factor MviM	0	95
SD1617_3340/	Enterotoxin	0	3
/	Enterotoxin	0	2
SD1617_0737/ilvB	Acetolactate synthase large subunit (EC 2.2.1.6)	81	476
SD1617_0939/ilvD	Dihydroxy-acid dehydratase (EC 4.2.1.9)	91	405
SD1617_0940/ilvA	Threonine dehydratase biosynthetic (EC 4.3.1.19)	59	380
SD1617_0942/ilvC	Ketol-acid reductoisomerase (EC 1.1.1.86)	133	268
SD1617_0938/ilvE	Branched-chain amino acid aminotransferase (EC 2.6.1.42)	58	225
SD1617_3738/ilvN	Acetolactate synthase small subunit (EC 2.2.1.6)	41	7
/	IlvBN operon leader peptide	0	687
SDY_2022/phoP	Transcriptional regulatory protein PhoP	222	472
SDY_2023/phoQ	Sensor histidine kinase PhoQ (EC 2.7.13.3)	136	117
SDY_3003/barA	Signal transduction histidine-protein kinase BarA (EC 2.7.13.3)	24	13
SDY_1104/uvrY	BarA-associated response regulator UvrY (= GacA = SirA)	261	314
SDY_2892/csrA	Carbon storage regulator	1021	662
SD1617_4387/hisF	Imidazole glycerol phosphate synthase cyclase subunit (EC 4.1.3)	31	189
SD1617_4390/hisB	Histidinol-phosphatase (EC 3.1.3.15)/imidazoleglycerol-phosphate dehydratase (EC 4.2.1.19)	28	186
SD1617_4391/hisC	Histidinol-phosphate aminotransferase (EC 2.6.1.9)	13	161
SD1617_4388/hisA	Phosphoribosylformimino-5-aminoimidazole carboxamide ribotide isomerase (EC 5.3.1.16)	28	132
SD1617_4392/hisD	Histidinol dehydrogenase (EC 1.1.1.23)	3	132
SD1617_4386/hisE	Phosphoribosyl-AMP cyclohydrolase (EC 3.5.4.19)/phosphoribosyl-ATP pyrophosphatase (EC 3.6.1.31)	0	96
SD1617_4393/hisG	ATP phosphoribosyltransferase (EC 2.4.2.17) - HisGl	5	399
SDY_2218/hisH	Imidazole glycerol phosphate synthase amidotransferase subunit (EC 2.4.2)	22	254
SD1617_4262/	Cold shock protein of CSP family - CspA (naming convention as in *E. coli*)	3991	2120
SDY_2381/cspD	Cold shock protein CspD	229	145
SDY_0546/cspE	Cold shock protein CspE	1714	222
SD1617_4774/	Cold shock protein of CSP family - CspC (naming convention as in *E. coli*)	0	2569
SDY_4448/groES	Heat shock protein 60 family co-chaperone GroES	3531	2405
SDY_4449/groEL	Heat shock protein 60 family chaperone GroEL	3994	3209
SDY_4172/ibpB	16 kDa heat shock protein B	146	91
SDY_4173/ibpA	16 kDa heat shock protein A	190	170
SDY_2787/grpE	Heat shock protein GrpE	491	409
	Heat shock protein C	6	15
SDY_3677/hslO	33 kDa chaperonin (Heat shock protein 33) (HSP33)	274	133
SD1617_5932/dcm	DNA-cytosine methyltransferase (EC 2.1.1.37)	0	117
SDY_4150/uhpA	Transcriptional regulatory protein UhpA	17	55
SDY_4659/creB	Response regulator CreB of two-component signal transduction system CreBC	42	25
SDY_4658/creA	Conserved uncharacterized protein CreA	120	122
SDY_4477/evgA	Positive transcription regulator EvgA	176	41
SDY_4478/evgS	Hybrid sensory histidine kinase in two-component regulatory system with EvgA	12	12
SDY_3723/hydH	Sensor protein of zinc sigma-54-dependent two-component system	68	54
SDY_3722/hydG	Response regulator of zinc sigma-54-dependent two-component system	76	40
SDY_1275/narL	Nitrate/nitrite response regulator protein NarL	66	19
SDY_1276/narX	Nitrate/nitrite sensor protein NarX	31	34
SDY_3874/glnL	Nitrogen regulation protein NtrB (EC 2.7.13.3)	69	67
SDY_3875/glnG	Nitrogen regulation protein NtrC	82	106
SDY_3214/ygiX	Two-component system response regulator QseB	25	33
SDY_3213/qseC	Sensory histidine kinase QseC	21	24
SDY_0856/rcsC	Sensor histidine kinase RcsC (EC 2.7.13.3)	32	32
SDY_0857/rcsB	DNA-binding capsular synthesis response regulator RcsB	870	899
SDY_1824/rstA	Transcriptional regulatory protein RstA	86	54
SDY_1825/rstB	Sensory histidine kinase in two-component regulatory system with RstA	60	56
SDY_2744/yfhA	Transcriptional response regulatory protein GlrR	44	41
SDY_2746/yfhK	Sensor histidine kinase GlrK	64	48
SDY_4443/dcuA	C4-dicarboxylate transporter DcuA	225	250
SDY_2186/baeR	Response regulator BaeR	53	34
SDY_2187/baeS	Sensory histidine kinase BaeS	6	1
SDY_1046/vsr	Very-short-patch mismatch repair endonuclease (Guanine–Thymine [G–T] specific)	0	28
SDY_1047/yedA	Uncharacterized innermembrane transporter YedA	0	0
SDY_1048/yedI	Innermembrane protein YedI	0	0
SDY_1970/	Uncharacterized protein YobF	30	2205

0: Not expressed.

### Virulence/invasion associated genes

*Shigella* virulence plasmid is an essential virulence determinant of the species and encodes the molecular machinery necessary for tissue invasion and intracellular survival. The virulence plasmid encodes the 30 kb Mxi-Spa type III secretion system (T3SS) and invasion plasmid antigens (Ipa proteins) required for invasion of the colonic and rectal epithelial cells and cell-to-cell spread of the bacteria, resulting in the symptoms of bacillary dysentery [17,18]. *Shigella* pathogenesis mainly relies on the Mxi-Spa T3SS and its effector proteins [19]. The invasion plasmid antigen (*ipa*H) gene, which was reported to be carried by all four *Shigella* species, was found to be highly expressed in invasive isolate in this study, whereas *ipa*D, a host injection protein was expressed only in noninvasive isolate. Further, *ipg*A, B, C, D, F known to facilitate local invasion in to epithelial cells, were also expressed only in noninvasive isolates (Table 3). Therefore, the virulence plasmid is the key molecular signature of *Shigella* spp. pathogenesis and is fundamental for initiating infection and manipulating the immune response of the host [18].

PhoQ/PhoP is a two-component system that governs virulence and regulates several cellular activities in *Shigella* spp. [20]. In the present study, PhoP was highly expressed in invasive isolate, whereas PhoQ showed no significant difference in the expression level. In addition, BarA-UvrY two-component system was shown to have increased expression in invasive isolate. This system also controls the activity of CsrA (carbon storage regulator) protein which regulates carbon metabolism, flagellar biosynthesis and biofilm formation. This process has been previously reported in uropathogenic *E. coli* [21]. We observed that CsrA protein was upregulated in noninvasive isolate.

### Stress-associated genes

Bacteria have developed a number of mechanisms to adapt the changing environmental conditions within the cells. One such mechanism is the production of small cold shock proteins (Csp) to counteract the sudden temperature downshift. Csps have been shown to contribute to osmotic, oxidative, starvation, pH and ethanol stress tolerance as well as to host cell invasion [22]. CspA is a major cold shock protein, first described in *E. coli* [22] was found to have significant differences in the expression level between the invasive and noninvasive isolate. Similarly, CspD and CspE proteins showed significant differences in their expressions, whereas CspC was highly and solely expressed in invasive isolate (Table 3). Another defense mechanism against various environmental stresses is the production of heat shock proteins. Heat shock proteins that are important for cell survival and are usually related to the virulence of the pathogens have been expressed in both the isolates [23].

### Genes involved in metabolism

In this study, *ilv* proteins such as *ilv*A, B, C, D, E and N involved in amino acid biosynthesis showed significantly increased expression in invasive isolate. Histidine (*his*) proteins like *his*A, B, C, D, E, F, G and H were found to have significant upregulation in invasive isolate. Further, member of the two-component regulatory system NtrB/NtrC and other regulator proteins like NarL and NarX involved in the regulation of nitrogen was expressed in both the isolates with no significant difference in the expression level. Similarly, several other genes such as (*ygi*X, *qse*C, *rcs*C, *rcs*B, *rst*A, *rst*B, *yfh*A, *yfh*K, *dcu*A, *bae*R, *bae*S, *vsr*) were present but showed no significant difference between the isolates.

### Cellular process & signaling

During *Shigella* infection, certain effector proteins promote cell survival. IpgD which associated with increased intracellular bacterial replication [24] was highly and solely expressed in noninvasive isolate as expected. Further *osp*C and *vir*A were also found to be expressed in noninvasive isolate [24]. DNA methylation is an important component in numerous cellular processes and plays an important role in regulating gene expression [25,26]. DNA cytosine methyltransferase protein was only slightly expressed in invasive isolate in this study.

### Uncharacterized genes

Two genes encoding uncharacterized proteins were identified. Uncharacterized innermembrane transporter *Yed*A gene was not expressed in the study isolates, which has been previously identified as hypothetical protein in *S. dysenteriae* strain Sd197. Another gene named *Yob*F, which is a small protein with no known function showed significantly increased expression in invasive isolate. Yet the functions of these genes remain obscure.

## Conclusion & future perspective

*Shigella* spp. is a highly contagious pathogen and humans are the only reservoir that spreads through fecal–oral contamination. The invasive ability of this pathogen is a key determinant in the establishment of the disease. The invasive phenotype of *Shigella* spp. is linked to the expression of various effector/regulatory genes. The differential protein expression by *S. dysenteriae* serotype 9 observed in this study suggests that it has a specific response to particular intracellular environment. Notably, many uncharacterized genes with unknown functions demonstrate the complexity of the regulatory network in *S. dysenteriae*. These genes needs to be further characterized to understand unidentified strategies for infection and successful survival of this pathogen. Further, the *in vivo* mechanism of *S. dysenteriae* invasion are difficult to fully study until the intracellular environment is mimicked *in vitro*. To the best of our knowledge, this is the first Indian study that compares the gene expression profile of clinical *S. dysenteriae* serotype 9 with respect to their invasion.

**Executive summary**

- Most of the earlier studies on mechanisms underlying pathogenesis was derived from *Shigella flexneri*. However, the present study shows the invasion process of *Shigella dysenteriae* serotype 9.
- RNA sequencing was done to study the differential expression of genes involved in the invasion process of the pathogen with the respect to the infection site.
- On virulence analysis, enterotoxin gene (*set*) and invasion associated genes such as *ipa*H and *ial* was identified in two, one and three isolates, respectively.
- For antimicrobial resistance, only two isolates harbored genes that codes for β-lactams, trimethoprim/sulfamethoxazole, fluroquinolones and cephalosporins resistance.
- RNA-Seq analysis showed significant fold change between noninvasive Sd_FC3355 and invasive Sd_BA42767 strains for the genes involved in invasion, virulence, motility and other cellular processes.
- Majority of the genes (44/56 genes) were highly expressed in noninvasive isolate, which is the primary site of invasion for *Shigella* spp. Few genes were expressed only in invasive isolate Sd_BA42767, which shows the significance of these genes in the invasive phenotype of the strain.
- This study explores that *Shigella* spp. has a specific response to particular intracellular environment.
- The identification of genes with uncharacterized functions demonstrates the complexity of the regulatory network in *S. dysenteriae*.
